# Triple and double twin interfaces in magnesium—the role of disconnections and facets

**DOI:** 10.1038/s41598-023-30880-w

**Published:** 2023-03-08

**Authors:** Martina Ruffino, John Nutter, Xun Zeng, Dikai Guan, W. Mark Rainforth, Anthony T. Paxton

**Affiliations:** 1 Department of Physics, King’s College London, London, WC2R 2LS UK; 2 Department of Materials Science and Engineering, University of Sheffield, Sheffield, S1 3JD UK; 3 Department of Mechanical Engineering, University of Southampton, Southampton, SO17 1BJ UK; 4 Department of Materials, Imperial College London, London, SW7 2AZ UK

**Keywords:** Surfaces, interfaces and thin films, Transmission electron microscopy, Surfaces, interfaces and thin films

## Abstract

Twin boundaries have been shown to deviate from the twinning planes in hcp metals, and facets have often been observed in twin interfaces. This study presents a twinning disconnection-based model for faceting in single, double and triple twin boundaries in magnesium. Primary twinning disconnections predicted via symmetry arguments are shown to produce commensurate facets in single twin boundaries, which are then transformed into commensurate facets in double twin boundaries via the action of secondary twinning disconnections. In contrast, it is shown that for triple twin boundaries with tension-compression-tension twinning sequence, no commensurate facets can be produced by the action of tertiary twinning disconnections. The effect of facets on the macroscopic orientation of twin interfaces is discussed. Theoretical findings are validated by a transmission electron microscopy study of a hot rolled Mg-1.18wt%Al-1.77wt%Nd alloy. Single and double twins are observed, as well as rare triple twins, and the interface between the matrix and a triple twin is captured for the first time. Facets consistent with theoretical predictions are imaged via high-resolution TEM and macroscopic deviations of the boundaries from the primary twinning planes are measured.

## Introduction

In hexagonal close packed (hcp) metals, twinning is the main route for the accommodation of plastic deformation together with slip at all temperatures^1,2^. In magnesium, whose axial ratio *q* is 1.624, twinning occurs on the $\{10\bar{1}2\}$, $\{10\bar{1}1\}$ and $\{10\bar{1}\bar{3}\}$ planes (the last being the reciprocal mode of the $\{10\bar{1}1\}$ mode)^3^. However, twinning on planes other than these was reported as early as 1956^4^ and 1957^5^, and it was later proposed that these modes could be attributed to successive twinning events, or double twinning^6^. Since then, double twins have been observed repeatedly in hcp metals with the aid of electron microscopy, with $\{10\bar{1}1\}-\{10\bar{1}2\}$ double twins being the most common^7–11^, though $\{10\bar{1}2\}-\{10\bar{1}1\}$^12^ and $\{10\bar{1}2\}-\{10\bar{1}2\}$^13^ double twins have also been reported. Theoretical predictions of the habit planes of double twins have not found full confirmation in experiment; however, consistency has eluded single twin boundaries as well. Deviations of the interfaces from the twinning planes of hcp metals have been reported in $\{10\bar{1}2\}$^14^ and $\{10\bar{1}1\}$^15^ twins. It was suggested that these changes in orientation could be attributed to the presence of facets in the boundaries^16^, and indeed facets have been observed in several instances in hcp metals^14,17,18^. One can then ask what becomes of these facets in the double twin boundaries, and in what measure their presence might determine the macroscopical orientation of the interfaces. Therefore, in the present work we approach the problem of facets first from a theoretical point of view, analysing the kinds of facets that can arise in single and double twin boundaries as pileups of admissible twinning defects predicted by Pond’s topological model of interfacial defects^19^. Theoretical predictions are then validated by experimental observations obtained via high-resolution transmission electron microscopy (HRTEM): several twin-like objects and their boundaries are investigated in a Mg-1.18wt%Al-1.77wt%Nd alloy, revealing the presence of single and double twins, as well as rarely observed triple twins. Notably, previously unrevealed interfaces between triple twins and the matrix are observed.

## Theory: twinning disconnections and facets

Twinning is described as a simple shear occurring on an invariant plane ${\textrm{K}}_1$ along the shear direction $\pmb {\eta }_1$. A second plane ${\textrm{K}}_2$ is left undistorted and the plane of shear S is that containing the normals to ${\textrm{K}}_1$ and ${\textrm{K}}_2$, as well as $\pmb {\eta }_1$; the conjugate shear direction $\pmb {\eta }_2$ is the intersection between ${\textrm{K}}_2$ and S^2^. Classically, the original sites are restored in the twin in a different orientation, i.e. the parent crystal and the twin are related by a rotation. However, when the primitive unit cell contains more than one atom, not all sites are shifted to the correct positions by the shear transformation, and they must shuffle.The literature has extensively shown that twin growth is accomplished by the motion of twinning dislocations along the twin boundary^2^. Recently, Pond et al.^20^ proposed that a dislocation-based description of the growth mechanism for $(10\bar{1}2)$ twinning in hcp metals can account for both the shear and shuffle parts of the total transformation. In this model, the motion of twinning disconnections (so termed as they possess both dislocation and step character^21^) accomplishes the twinning transformation: the Burgers vector of the disconnection is responsible for the shear part of the transformation, and the step for the atomic shuffles.

**Figure 1:**
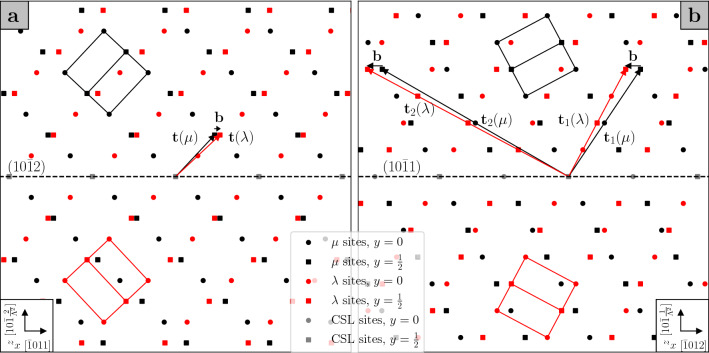
Dichromatic patterns for the $(10\bar{1}2)$ (**a**) and $(10\bar{1}1)$ (**b**) twins in magnesium, $q=1.624$, projected onto the plane of shear. $\mu$ sites, in black, belong to the matrix, and $\lambda$ sites, in red, belong to the twinned lattice. Overlapping black and red sites are given in grey, and they constitute the coincident site lattice (CSL).

Admissible twinning disconnections for a given twin can be obtained via the topological model of interfacial defects^19,22,23^. This model is based on the analysis of the symmetry group of a bicrystal, i.e. a composite of two crystals, conventionally labelled $\mu$ and $\lambda$, related by a transformation $\textbf{P}$. The operation characterising a given defect in a bicrystal interface is obtained by combining symmetry operations that are present in the symmetry groups of the $\mu$ and $\lambda$ crystals, but are broken in the bicrystal. In this framework, a twinning disconnection arises when translations of the $\mu$ and $\lambda$ crystal lattices are not coincident, and the resulting Burgers vector, given by the difference in the translation vectors $\textbf{t}(\mu )$ and $\textbf{t}(\lambda )$ expressed in the same coordinate system, $\textbf{b}=\textbf{t}(\lambda ) - \textbf{P}\textbf{t}(\mu )$, is parallel to the shear direction $\pmb {\eta }_1$. For a given ${\textrm{K}}_1$ plane normal $\hat{\textbf{n}}$, each of the translations $\textbf{t}(\lambda )$ and $\textbf{t}(\mu )$ is associated with a step of height $h_{\lambda }=\hat{\textbf{n}}\cdot \textbf{t}(\lambda )$ and $h_{\mu }=\hat{\textbf{n}}\cdot \textbf{t}(\mu )$ respectively, and the step height of the disconnection is taken to be the smaller of the two^21^; in the case of a twinning disconnection, the step heights corresponding to $\textbf{t}(\lambda )$ and $\textbf{t}(\mu )$ are equal, and either can be used as the step height for the disconnection. A useful graphical representation of the Burgers vectors of twinning disconnections can be obtained by constructing a dichromatic pattern, i.e. a schematic of the interpenetrating $\lambda$ and $\mu$ crystal lattices. Then, the Burgers vectors of admissible defects connect $\lambda$ and $\mu$ sites, and the step heights are easily visualised as well. Examples are given for the $(10\bar{1}2)$ and $(10\bar{1}1)$ twins in magnesium in Fig. 1.

### Faceting in $(10\bar{1}2)$ and $(10\bar{1}1)$ twin boundaries

**Figure 2:**
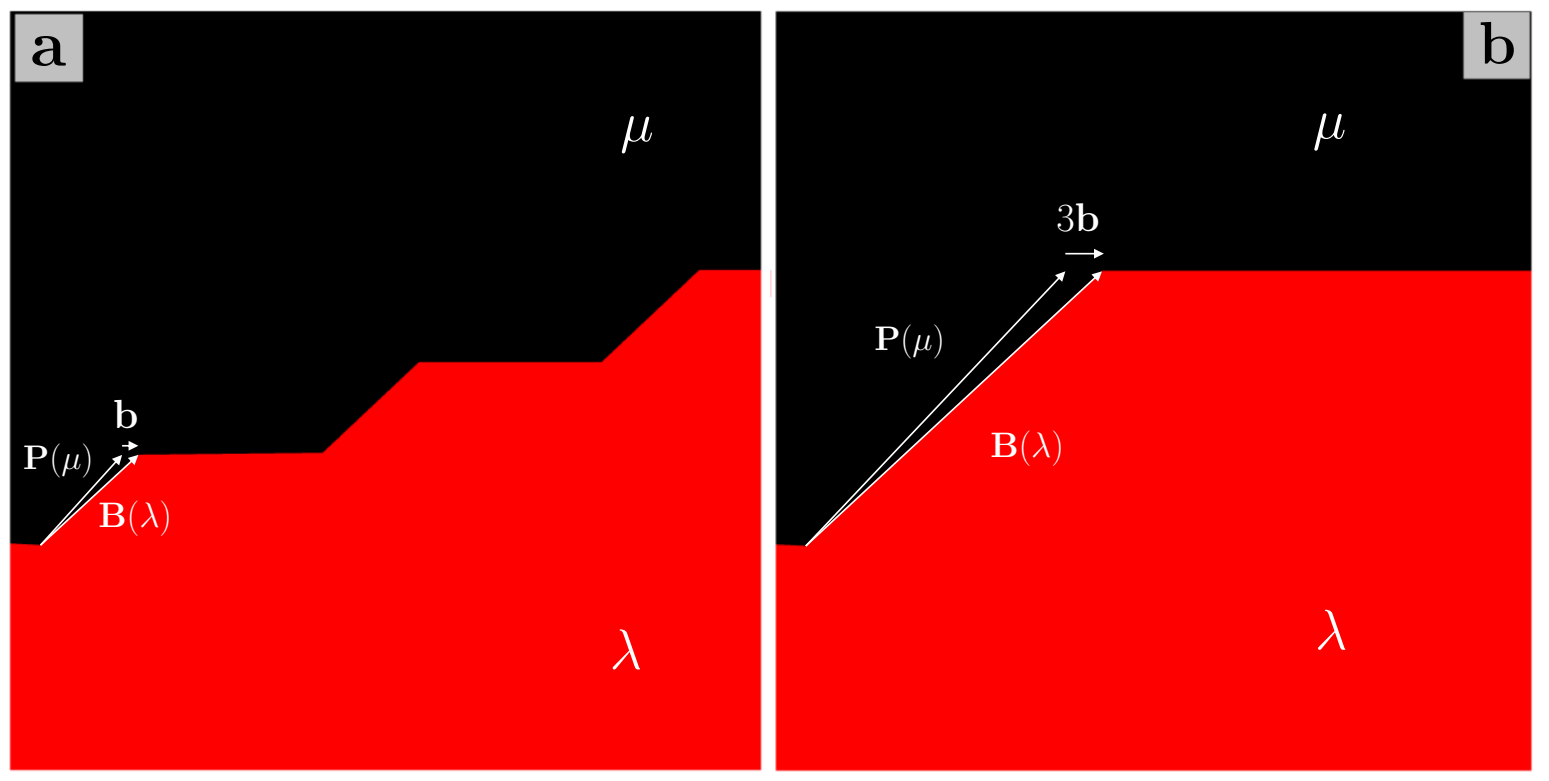
Pileup of twinning disconnections leading to the formation of a PB facet in a $(10\bar{1}2)$ twin boundary. The parent crystal ($\mu$) is represented in black, and the twinned crystal ($\lambda$) is represented in red.

For each of the twinning modes occurring in magnesium, the transformation relating the $\mu$ and $\lambda$ crystals can be described as a rotation of $180^{\circ }$ about the shear direction $\pmb {\eta }_1$, such that $\textbf{P}=\textbf{R}$. Constructing the dichromatic pattern for the $(10\bar{1}2)$ twin (Fig. 1a), one finds that the Burgers vector of the twinning disconnection associated with this mode is given by $\textbf{b}=\frac{2-\Lambda ^2}{2+\Lambda ^2}[\bar{1}011]$, where $\Lambda =q\sqrt{2/3}$ and *q* is the *c*/*a* ratio, and the disconnection has step height $h=2d_{(10\bar{1}2)}$, where $d_{(10\bar{1}2)}$ is the interplanar spacing of $(10\bar{1}2)$ planes^24^. It is evident from the dichromatic pattern that an infinite number of pairs of translation vectors from the $\lambda$ and $\mu$ crystal lattices produce the required Burgers vector, but if $\textbf{t}(\mu )=[000\bar{1}]$ and $\textbf{t}(\lambda )=[10\bar{1}0]$ are chosen, then it can be seen that these vectors lie in the prismatic (P) plane of the $\mu$ crystal and the basal (B) plane of the $\lambda$ crystal respectively^25^. Therefore, the presence of such a disconnection in the twin boundary results in the coalescence of the prismatic plane of the matrix ($\mu$) and the basal plane of the twin ($\lambda$), and a small PB facet is created^26^, with the disconnection accommodating the mismatch between the basal and prismatic planes^27^. Similarly, a BP facet can be created by taking $\lambda$ as the matrix and $\mu$ as the twin. It should be noted that the notation adopted to label the facets in the text of this section is the following: the plane of the matrix is given first, and then that of the twin, such that e.g. BP stands for ${\textrm{B}}^{\textrm{matrix}}{\textrm{P}}^{\textrm{twin}}$. Simulations have suggested that the accumulation of such disconnections in the $(10\bar{1}2)$ twin boundary may be responsible for the formation of longer BP and PB facets that are often observed experimentally^27–29^, with the formation energy of such facets being comparable to that of the twin boundary, thus pointing towards the stability of the facets^27^. A schematic illustrating such a pileup of disconnections in a $(10\bar{1}2)$ twin boundary is diplayed in Fig. 2. This description of BP and PB facets also provides a mechanism for facet migration, via the emission of single twinning disconnections that glide on the $(10\bar{1}2)$ plane, as opposed to the movement of long facets as wholes, such that it would be misleading to identify the facet as a “superdisconnection”^27^. Alternatively, BP and PB facets have been described as disclination dipoles located at the junctions between the facet and the twin boundary^29–32^.

Similarly, the emergence of facets can be predicted in the $(10\bar{1}1)$ twin boundary. As is observed from the dichromatic pattern for this twin (Fig. 1b), the twinning disconnection associated with shear on the $(10\bar{1}1)$ plane has Burgers vector $\textbf{b}=\frac{2\Lambda ^2-3}{2\Lambda ^2+1}[10\bar{1}\bar{2}]$ and step height $h = 4d_{(10\bar{1}1)}$, with $d_{(10\bar{1}1)}$ the interplanar spacing of $(10\bar{1}1)$ planes^33,34^. In pair-potential simulations this disconnection has been found to have low mobility on the $(10\bar{1}1)$ plane, and it has been pointed out that a disconnection with step height $h=2d_{(10\bar{1}1)}$ and Burgers vector $\textbf{b} = \frac{1}{3(2\Lambda ^2+1)}\pmb {\eta }_1$, $\pmb {\eta }_1 = [(5-2\Lambda ^2),-(2\Lambda ^2+1),4(\Lambda ^2-1),3(2\Lambda ^2-3)]$, has lower core energy as well as higher mobility^34^. However, this disconnection has an additional screw component and does not shear the crystal along the experimentally observed direction, i.e. $[\bar{1}012]$. Wang et al.^35^ proposed that the 4-layer disconnection could be regarded as a superposition of two 2-layer disconnections with screw components of alternating sign, which would then restore the required shear direction; but if one of the two 2-layer disconnections were favoured by the shear stress, its prevalence would once again lead to an irrational shear direction. In light of these considerations, we discuss faceting in the $(10\bar{1}1)$ twin boundary in relation to the 4-layer disconnection with rational $\pmb {\eta }_1$.

From the dichromatic pattern for the $(10\bar{1}1)$ twin, one can surmise that there are two kinds of facets that can form in this boundary. Taking $\textbf{t}_1(\mu )=[\bar{1}01\bar{2}]$ and $\textbf{t}_1(\lambda )=[20\bar{2}0]$, it is easily seen that $\textbf{t}_1(\mu )$ lies in the first pyramidal plane (Py, $(\bar{1}011)$) of the matrix, and $\textbf{t}_1(\lambda )$ lies in the basal plane of the twin; hence, the resulting disconnection brings the Py and B planes into coincidence, forming a small PyB facet^26,32^, while a BPy facet will be obtained by swapping the matrix and the twin. An accumulation of such twinning disconnections behind an obstacle can thus lead to the formation of longer facets. The second kind of facet for this twin boundary is obtained by taking $\textbf{t}_2(\mu )=[30\bar{3}\bar{2}]$ and $\textbf{t}_2(\lambda )=[000{4}]$, such that $\textbf{t}_2(\mu )$ and $\textbf{t}_2(\lambda )$ lie respectively in the third pyramidal plane of the matrix ($(10\bar{1}3)$, 3Py), and the prismatic plane of the twin. The resulting Burgers vector is the same as that yielded by $\textbf{t}_1(\mu )$ and $\textbf{t}_1(\lambda )$, but a different facet is formed, i.e. a 3PyP facet, or P3Py if matrix and twin are swapped^32^.

The facets described above have been observed in many experimental works. In particular, BP and PB facets in $(10\bar{1}2)$ twin boundaries have been widely reported in TEM studies of magnesium alloys and cobalt^14,18,36^; BPy, PyB, P3Py and 3PyP facets were found in $(10\bar{1}1)$ twin boundaries in titanium^16^, magnesium^15,17^ and cobalt^37^. In many cases, the macroscopic twin boundaries were reported to deviate from the ${\textrm{K}}_1$ planes by significant amounts^14–16^; this can be attributed to the presence of facets. It is then natural to ask how these facets may affect the appearance of double twin boundaries, which often arise in magnesium alloys. We examine this question in the next section.

### Faceting in double twin boundaries

Double twinning is described as the internal (secondary) retwinning on a secondary ${\textrm{K}}_1$ plane of an initial (primary) twin. A theory of double twinning in hexagonal metals was first proposed by Crocker^6^, who postulated that the combination of two simple shears leading to double twinning must produce a total transformation which is itself a simple shear. His analysis was then absorbed into a more general theory of non-classical twinning^38^, where double twinning was cited as a special case. Despite Crocker’s and his successive coworkers’ mathematical descriptions of double twinning as an equivalent single twinning mode, experimental evidence shows that double twins are produced by successive twinning events, and more recent theories of double twin formation have focused on lattice dislocations impinging on primary twin boundaries as sources of secondary twinning disconnections^39^. These would then go on to nucleate the secondary twin within the primary twin. In the present study we focus on $\{10\bar{1}1\}-\{10\bar{1}2\}$ (compression-tension) and $\{10\bar{1}2\}-\{10\bar{1}1\}$ (tension-compression) double twins, henceforth referred to as C-T and T-C double twins respectively.

Due to hexagonal symmetry, each single twinning event can occur on one of six equivalent ${\textrm{K}}_1$ planes; this means that there are 36 double twin variants for each double twinning sequence. However, only four of these variants are geometrically distinct, and they are classified as types 1-4 (we will adhere to the usage of “type”^8^ for the four double twins although it clashes with the four types of classical twin.^2^): for types 1 and 2, the primary and secondary twin share the plane of shear, resulting in rational misorientation relations, while in types 3 and 4 the two twinning modes have different planes of shear, leading to irrational misorientation relations. The misorientation relations describe the relationship between the coordinate systems of the matrix and the twinned (or doubly twinned) crystal, i.e. $\textbf{M}_{\textrm{tot}}=\textbf{O}_{\textrm{f}}\textbf{O}_{\textrm{i}}^{-1}$, where $\textbf{O}_{\textrm{i}}$ and $\textbf{O}_{\textrm{f}}$ are the operations representing the initial and final orientations respectively, i.e. the matrix and the twin or double twin^8,10^. For a double twin, we take the orientation of the matrix to be represented by the identity matrix, $\textbf{O}_{\textrm{i}}=\textbf{I}$, and the final orientation to be the result of successive rotations ascribed to each twinning event; these are rotations of $180^{\circ }$ about the respective shear directions, expressed in their own coordinate systems, and are termed $\textbf{R}_{\textrm{A}}$ and $\textbf{R}_{\textrm{B}}$. Therefore, the total transformation, expressed in the coordinate system of the matrix, is given by $\textbf{M}_{\textrm{AB}}=\textbf{R}_{\textrm{A}}\textbf{R}_{\textrm{B}}\textbf{R}_{\textrm{A}}^{-1}\textbf{R}_{\textrm{A}} = \textbf{R}_{\textrm{A}}\textbf{R}_{\textrm{B}}$. Misorientation relations are often given as minimum axis/angle pairs, i.e. as rotations about an axis: these can be extracted from $\textbf{M}_{\textrm{AB}}$, which is itself a rotation. Confining ourselves to double twin variants whose component twinning events have a common plane of shear, we see that misorientation relations for these cases can be represented as rotations about the normal to the plane of shear, i.e. $[1\bar{2}10]$. Moreover, one can show that the axis/angle pairs yielded by C–T and T–C double twins are the same, even though $\textbf{R}_{\textrm{A}}\textbf{R}_{\textrm{B}}\ne \textbf{R}_{\textrm{B}}\textbf{R}_{\textrm{A}}$ in general: the rotation angle $\theta$ is given by $\cos \theta =\frac{1}{2}({\textrm{Tr}}(\textbf{M}_{\textrm{AB}})-1)$, and the trace of a matrix product is invariant under cyclic permutations, in this case ${\textrm{Tr}}(\textbf{R}_{\textrm{A}}\textbf{R}_{\textrm{B}})={\textrm{Tr}}(\textbf{R}_{\textrm{B}}\textbf{R}_{\textrm{A}})$. The axis/angle pairs of interest calculated so are listed for magnesium in Table 1.

**Table 1:** Labels and axis/angle pairs of some magnesium twinning modes, $q=1.624$.

${\textrm{K}}_1$ plane(s)	Label	Axis/angle
$(10\bar{1}2)$	Tension (T)	$86^{\circ }[1\bar{2}10]$
$(10\bar{1}1)$	Compression (C)	$56^{\circ }[1\bar{2}10]$
$(10\bar{1}1)-(10\bar{1}2)$	C–T type 1	$38^{\circ }[1\bar{2}10]$
$(10\bar{1}1)-(\bar{1}012)$	C–T type 2	$30^{\circ }[1\bar{2}10]$
$(10\bar{1}2)-(10\bar{1}1)$	T–C type 1	$38^{\circ }[1\bar{2}10]$
$(10\bar{1}2)-(\bar{1}011)$	T–C type 2	$30^{\circ }[1\bar{2}10]$
$(10\bar{1}2)-(10\bar{1}1)-(10\bar{1}2)$	T-C-T type 1a	$48^{\circ }[1\bar{2}10]$
$(10\bar{1}2)-(10\bar{1}1)-(\bar{1}012)$	T-C-T type 1b	$56^{\circ }[1\bar{2}10]$
$(10\bar{1}2)-(\bar{1}011)-(10\bar{1}2)$	T-C-T type 2a	$56^{\circ }[1\bar{2}10]$
$(10\bar{1}2)-(\bar{1}011)-(\bar{1}012)$	T-C-T type 2b	$64^{\circ }[1\bar{2}10]$

**Figure 3:**
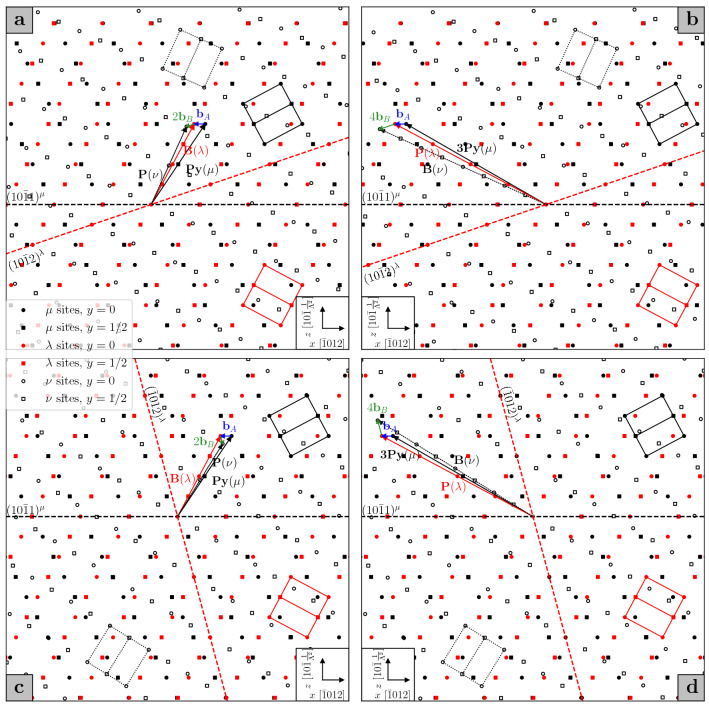
Compression-tension (C–T) double twin facets for types 1 (**a**) and (**b**) and 2 (**c**) and (**d**), $q=1.624$, projected onto the plane of shear, common to the two twinning mechanisms. $\mu$ sites, in black, belong to the matrix; $\lambda$ sites, in red, belong to the primary twin; $\nu$ sites, in white, belong to the secondary twin. The hexagonal unit cells are similarly drawn for each lattice. The translation vectors that define the disconnections are denoted by the names of the planes they lie in, in order to highlight the kinds of facets they can form.

**Figure 4:**
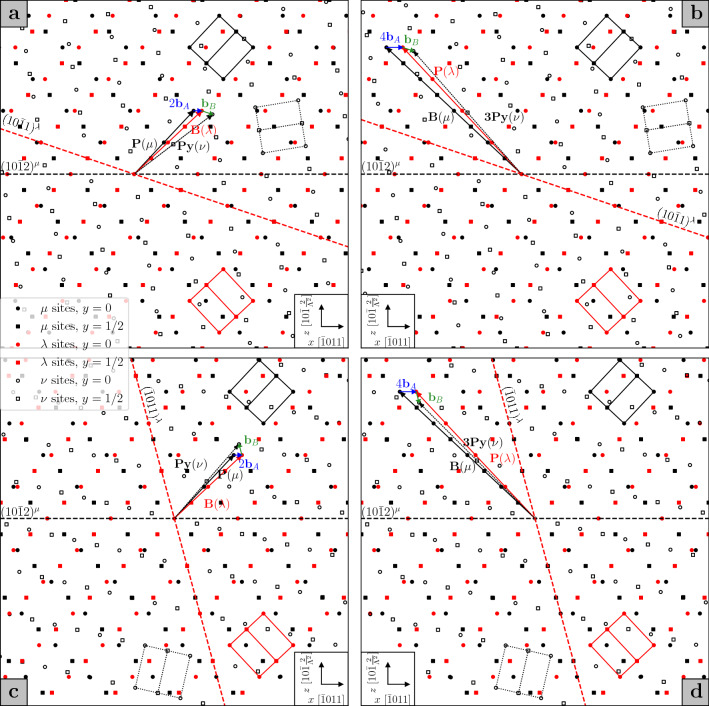
Tension-compression (T–C) double twin facets for types 1 (**a**) and (**b**) and 2 (**c**) and (**d**), $q=1.624$, projected onto the plane of shear, common to the two twinning mechanisms. $\mu$ sites, in black, belong to the matrix; $\lambda$ sites, in red, belong to the primary twin; $\nu$ sites, in white, belong to the secondary twin. The hexagonal unit cells are similarly drawn for each lattice. The translation vectors that define the disconnections are denoted by the names of the planes they lie in, in order to highlight the kinds of facets they can form.

Having described the orientation of the doubly twinned crystal with respect to the matrix, we now consider how the primary twin boundary is affected by the secondary twinning event. Crocker’s theory^6^ focused on the identification of an invariant plane on which the double twinning shear would occur in the event of a double twin nucleus growing within the matrix. However, if the view is taken that the secondary twin nucleates inside an already formed primary twin, the resulting habit plane is unlikely to correspond to the invariant plane predicted by Crocker. Indeed, measured matrix to double twin interface planes have been found to deviate from the primary ${\textrm{K}}_1$ planes by only a few degrees^7^, producing irrational boundary planes that are approximated by high indices. In order to propose an explanation, we may turn to the problem of facets. We have shown how facets may exist in single twin boundaries, such that it is reasonable to assume that they will be present in both the primary twin boundary and the internal boundary between the primary and the secondary twin. As the secondary twin grows, its facets will disintegrate into the component disconnections, which then glide towards the primary twin boundary. When the secondary twinning disconnections reach a preexisting facet on the primary twin boundary, formed by primary twinning disconnections, they may transform the facet into a commensurate double twin facet. This can be illustrated with the aid of Figs. 3 and 4.

We start from the C–T type 1 double twin, Fig. 3a–b. In order to illustrate the transformations that lead to the double twin orientation, a trichromatic pattern is constructed, where three sets of lattice points are drawn: sites $\mu$ represent the matrix, sites $\lambda$ the primary twin, obtained via a rotation $\textbf{R}_{\textrm{A}}$, and sites $\nu$ the secondary twin, produced by rotating $\mu$ sites by $\textbf{R}_{\textrm{A}}\textbf{R}_{\textrm{B}}$. The primary ${\textrm{K}}_1$ plane $(10\bar{1}1)^{\mu }$ and the secondary ${\textrm{K}}_1$ plane $(10\bar{1}2)^{\lambda }$ are also drawn. One can then depict the Burgers vector of the primary twinning disconnection as in Fig. 1b, choosing the 4-layer disconnection whose $\textbf{t}(\mu )$ and $\textbf{t}(\lambda )$ lie respectively in the first pyramidal plane of the matrix and basal plane of the primary twin, thus forming a PyB facet; the Burgers vector of this disconnection is labelled $\textbf{b}_{\textrm{A}}$, Fig. 3a. Subsequently, the $\lambda$ site sheared into position by the primary shear is moved by the secondary shear into a $\nu$ site, and by comparing Fig. 3a with Fig. 1a, one can see that this is accomplished by two secondary twinning disconnections with Burgers vector $\textbf{b}_{\textrm{B}}=\frac{2-\Lambda ^2}{2+\Lambda ^2}[10\bar{1}\bar{1}]^{\lambda }$. The translation vectors $\textbf{t}(\lambda )$ and $\textbf{t}(\nu )$ from which $\textbf{b}_{\textrm{B}}$ is obtained lie respectively in the basal plane of the primary twin and in the prismatic plane of the secondary twin. Thus, a PyB facet in the primary twin boundary combines with a BP facet of the secondary twin to form a PyP facet in the double twin boundary as a result of the combination of two secondary twinning disconnections for every one primary twinning disconnection. The total Burgers vector that generates such a PyP facet is then given in the coordinate frame of the matrix by

1 $$\begin{aligned} \textbf{b}_{\textrm{PyP}}=\textbf{b}_{\textrm{A}}+2\textbf{R}_{\textrm{A}}\textbf{b}_{\textrm{B}}. \end{aligned}$$

On the other hand, one can choose the second set of translation vectors from Fig. 1b, so that $\textbf{t}(\mu )$ lies in the third pyramidal plane of the matrix and $\textbf{t}(\lambda )$ lies in the prismatic plane of the primary twin, and the resulting primary twinning disconnection forms a 3PyP facet, Fig. 3b. Then, four secondary twinning disconnections are needed to shear the $\lambda$ site to its position in the double twin lattice $\nu$, such that $\textbf{t}(\nu )$ lies in the basal plane of the secondary twin. A 3PyP facet from the primary twin and PB facet of the secondary twin are thus combined to produce a 3PyB facet in the double twin boundary, whose generating Burgers vector is given by

2 $$\begin{aligned} \textbf{b}_{\textrm{3PyB}}=\textbf{b}_{\textrm{A}} + 4\textbf{R}_{\textrm{A}}\textbf{b}_{\textrm{B}}. \end{aligned}$$

Hence, two kinds of commensurate facets can be formed in a type 1 C–T double twin boundary: PyP and 3PyB.

Similarly, facets can be derived for C–T double twin boundaries of type 2, Fig. 3c–d. In this case, the secondary ${\textrm{K}}_1$ plane is $(\bar{1}012)$, but the kinds of facets formed are the same as those obtained for the type 1 C–T double twin. If the primary twinning disconnection is taken to generate a PyB facet of the primary twin boundary, then two secondary twinning disconnections forming a BP facet on the internal secondary twin boundary will transform the initial facet into a PyP facet of the double twin, Fig. 3c. If instead a primary twinning disconnection forming a 3PyP facet in the boundary is present, four secondary twinning disconnections describing secondary PB facets will be needed to create a 3PyB facet in the double twin boundary, Fig. 3d.

It is now straightforward to extend the analysis of facets performed above to tension-compression (T–C) double twins. The primary twinning event now occurs on the $(10\bar{1}2)$ plane, while the secondary ${\textrm{K}}_1$ plane is either $(10\bar{1}1)$ (type 1) or $(\bar{1}011)$ (type 2) of the primary twin; the primary twinning disconnection, $\textbf{b}_{\textrm{A}}$, is the 2-layer disconnection of Fig. 1a. Considering first the type 1 T–C double twin, two kinds of commensurate facets can be created in the double twin boundary. The first, Fig. 4a, is a PPy facet, arising from the combination of two primary twinning disconnections and one secondary twinning disconnection with Burgers vector $\textbf{b}_{\textrm{B}}=\frac{2\Lambda ^2-3}{2\Lambda ^2+1}[\bar{1}012]^{\lambda }$, such that

3 $$\begin{aligned} \textbf{b}_{\textrm{PPy}}=2\textbf{b}_{\textrm{A}}+\textbf{R}_{\textrm{A}}\textbf{b}_{\textrm{B}}. \end{aligned}$$

The primary twinning disconnections correspond to PB facets in the primary twin boundary, while the secondary twinning disconnection forms a BPy facet in the internal secondary twin boundary. One can then see that this is the same kind of facet created in a type 1 C–T double twin, only the order is reversed: in this case, the facet brings into coincidence the prismatic plane of the matrix and the first pyramidal plane of the secondary twin. Similarly, the second kind of T–C type 1 double twin facet is a B3Py facet, Fig. 4b, produced by the combination of four primary twinning disconnections (BP facet in the primary boundary) and one secondary twinning disconnection (P3Py facet in the internal secondary twin boundary), such that

4 $$\begin{aligned} \textbf{b}_{\textrm{B3Py}}=4\textbf{b}_{\textrm{A}}+\textbf{R}_{\textrm{A}}\textbf{b}_{\textrm{B}}. \end{aligned}$$

It then follows that similar facets can be encountered in the boundary of a type 2 T–C double twin: a PPy facet (Fig. 4c) and a B3Py facet (Fig. 4d).

The above study of faceting in double twin boundaries can help shed some light on the macroscopic appearance of the boundaries themselves. As was explained in the previous section, faceting of single twin boundaries can conspicuously deviate the macroscopic orientation of the boundary plane from the ${\textrm{K}}_1$ plane. It is reasonable to propose that upon nucleation of a secondary twin within a primary twin, the segments of the primary twin boundary oriented along the primary ${\textrm{K}}_1$ plane are left relatively unchanged, as any secondary twinning disconnections impinging on them are unable to glide any further. However, secondary twinning disconnections encountering primary facets may produce facets in the double twin boundary, whose orientations differ from the original facets by a few degrees. The macroscopic orientation of the double twin boundary is then altered, by an amount that depends on the density and length of the facets. This might go towards explaining the different orientations measured for double twin boundaries of the same type: for instance, Cizek and Barnett^7^, studying $\{10\bar{1}1\}-\{10\bar{1}2\}$ double twins in a magnesium alloy, found that the orientations of such boundaries could vary between the $\{30\bar{3}4\}$, $\{10\bar{1}1\}$ and $\{20\bar{2}3\}$ planes.

### Triple twin boundaries

The number of sequential twinning events need not be limited to two. In principle, a twinned grain could retwin multiple times, a phenomenon that has sometimes been denoted as hierarchical twinning^40^. In the present section we focus on internal retwinning of tension-compression (T–C) double twins where the tertiary twin is a tension twin, i.e. T-C-T triple twins. Considering only triple twins whose component modes all share a common plane of shear, as was done in the previous section, the tertiary twin can grow on one of two planes, i.e. $(10\bar{1}2)$ and $(\bar{1}012)$. This is possible for each of the two T–C double twin modes presented earlier, type 1 $(10\bar{1}2)-(10\bar{1}1)$ and type 2 $(10\bar{1}2)-(\bar{1}011)$, such that there are four T-C-T triple twin modes; these are labelled in Table 1 and axis/angle pairs are given for magnesium, $q=1.624$.

Following from the analysis in the previous section, the view is taken that the tertiary twin grows within the secondary twin via the motion of tertiary twinning disconnections, which glide towards the fully formed double twin boundary. The facets present in the double twin boundary are PPy and B3Py, for both double twin types. The tertiary twinning disconnections are 2-layer disconnections of the kind in Fig. 1a, and they can form BP and PB facets in the internal boundary between the secondary and the tertiary twin. However, upon encountering the matrix to double twin interface facets, the tertiary twinning disconnections cannot form commensurate facets in the triple twin boundary, as no rational planes of the matrix and the double twin can be brought into coincidence by a combination of primary, secondary and tertiary twinning disconnections for this sequence of twinning modes. Therefore, interfaces between matrix and T-C-T triple twins are fully incommensurate.

To our knowledge, this hypothesis has not been tested in the experimental literature. Studies of tertiary T-C-T twins in magnesium reported the observation of grains where the primary twin has grown to consume either a large portion of the matrix^41,42^ or its near entirety^42–46^, with subsequent secondary and tertiary twins growing in thin lamellae within the primary twin. Consequently, boundaries between matrix and triple twin have not been observed.

## Experimental methods

The Mg alloy used in this study was prepared by direct chill casting of pure Mg, Al, Nd feed stock. The final composition, as determined using X-ray fluorescence, was Mg-1.18wt%Al-1.77wt%Nd. The melt was stirred under an Ar and 3% ${\textrm{SF}}_6$ atmosphere for 30 minutes at $720^{\circ }$C before casting into a boron nitride coated steel mold preheated to $680^{\circ }$C. The filled mold was quenched by being slowly lowered into a water bath. Homogenisation was carried out at $420^{\circ }$C before being machined into slabs with dimensions of 100 x 50 x 10 mm.

Slabs were hot rolled at $420^{\circ }$C with a 10–15% reduction per pass and an intermediate annealing step of 10 minutes between passes. After 11 passes the final thickness was 2.3 mm with a total strain of 0.77.

**Figure 5:**
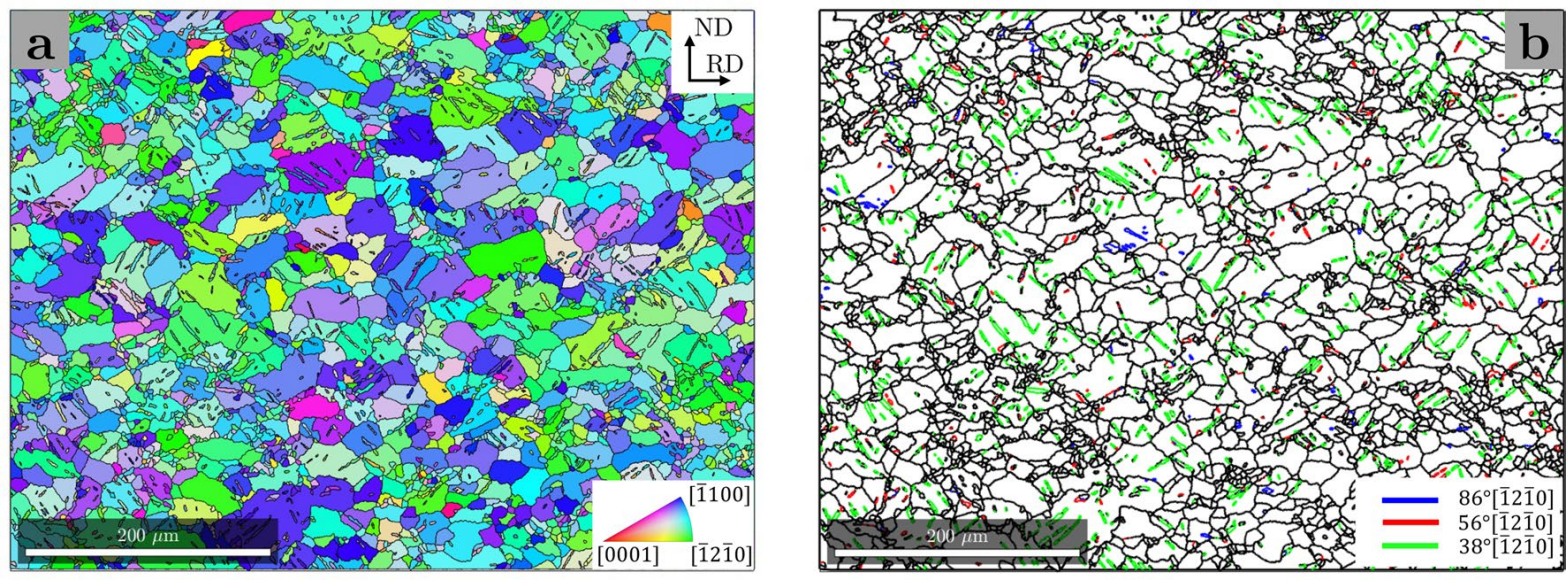
(**a**) EBSD IPS map of the microstructure of the processed Mg-1.18wt%Al-1.77wt%Nd alloy used in this study. (**b**) Boundaries exhibiting the tension twin (blue), compression twin (red) and C–T or T–C double twin (green) misorientation relations.

A preliminary study of the microstructure was carried out post processing using electron backscatter diffraction (EBSD) techniques. An EBSD inverse pole figure map of the sample in projection along the transverse direction is shown in Fig. 5a, along with a map of boundaries with misorientations corresponding to those of tension, compression and T–C or C–T “type 1” double twins in Fig. 5b. This figure shows that the sample contains a high density of double twins, thus ensuring its suitability for the investigation of double twin to matrix interfaces via a TEM study.

As a strong basal texture was detected in projection along the normal direction, the rolled slabs were sectioned parallel to the plane containing the rolling and transverse directions. TEM specimens were prepared by mechanically thinning the sections to approximately 100 $\mu$m followed by electropolishing in a Tenupol-5 twin jet electropolisher using a solution of 8.8 g Lithium Chloride, 18.6 g Manganese Perchlorate, 160 mL 2-butoxyethanol and 840 mL Methanol at $-35^{\circ }$C. TEM analysis was carried out using a JEOL JEM-F200 TEM operated at 200 kV. The samples were tilted down the $[1\bar{2}10]$ zone axis. Investigation of the HRTEM bright field images was aided by the careful comparison of images of the same areas obtained through Fourier filtering.

## Results

**Figure 6:**
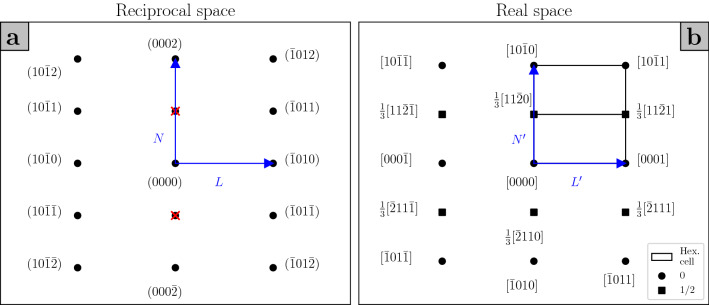
Relationship between reciprocal (**a**) and real space (**b**) projections along the $[1\bar{2}10]$ direction of the hexagonal lattice.

Investigation of selected area diffraction (SAD) patterns combined with observations of boundary planes in bright field images allowed us to identify multiple twinning events occurring in the same sample. In order to aid the interpretation of SAD patterns, it is useful to draw the reader’s attention to the relationship between the reciprocal and real space projections of the hexagonal lattice along the $[1\bar{2}10]$ direction, Fig. 6. Spots in Fig. 6a represent lattice planes in reciprocal space, with the distance between the spots and the origin corresponding to $1/d_{(hkil)}$, with $d_{(hkil)}$ the spacing of (*hkil*) planes; in Fig. 6b, circles and squares denote different layers of lattice sites as seen when looking down the $[1\bar{2}10]$ projection. It should be noted that $\frac{N}{L}=\frac{N'}{L'}$, such that the two projections are identical except for scaling. As a consequence, SAD patterns of regions containing crystals in different orientations can be indexed with the aid of dichromatic patterns of the bicrystals of interest, opportunely scaled.

**Figure 7:**
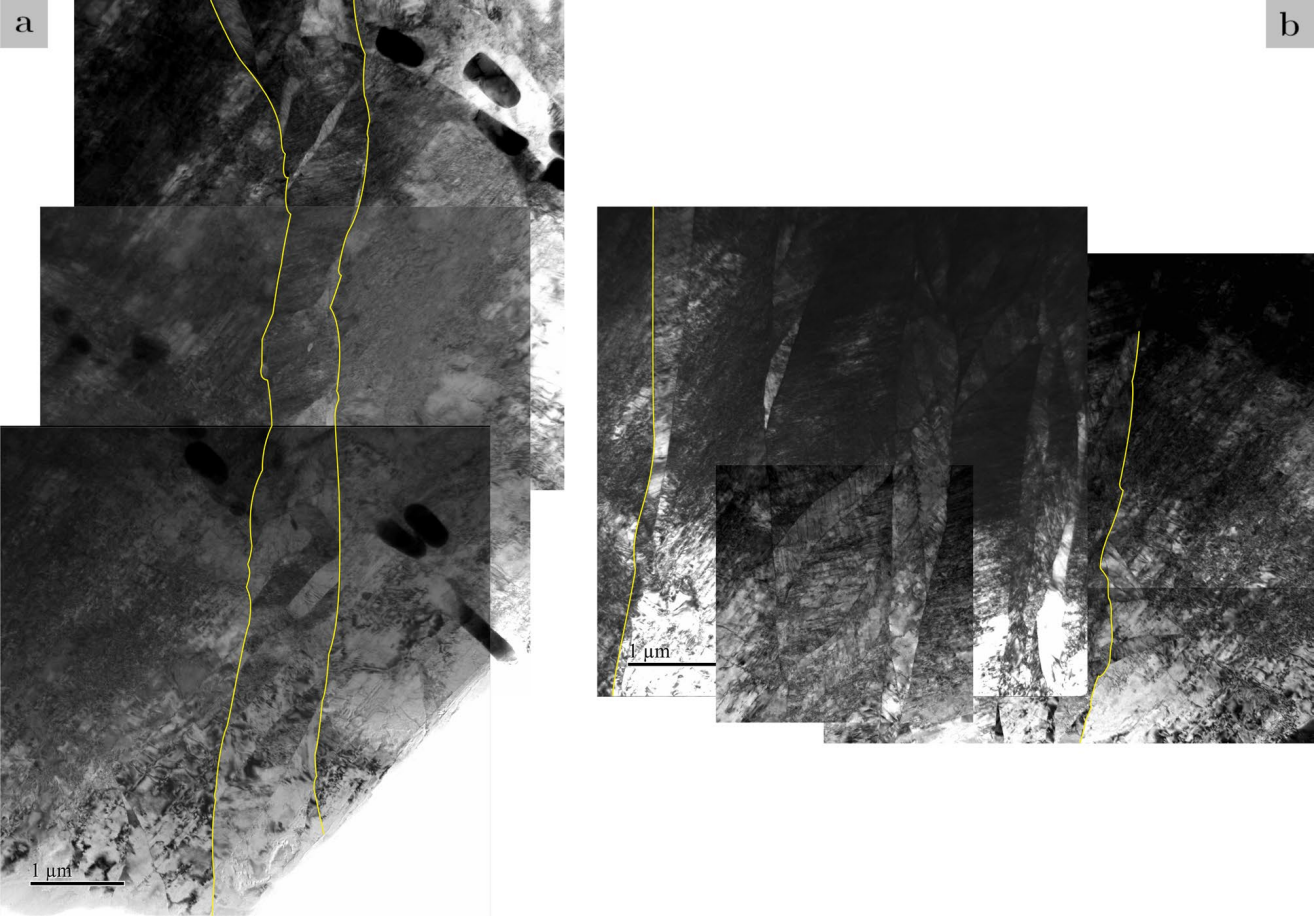
TEM Bright field images of two areas of interest identified in the sample: (**a**) a long, narrow twin-like object; (**b**) a cluster of twins. The contours of the two objects were delineated for clarity.

**Figure 8:**
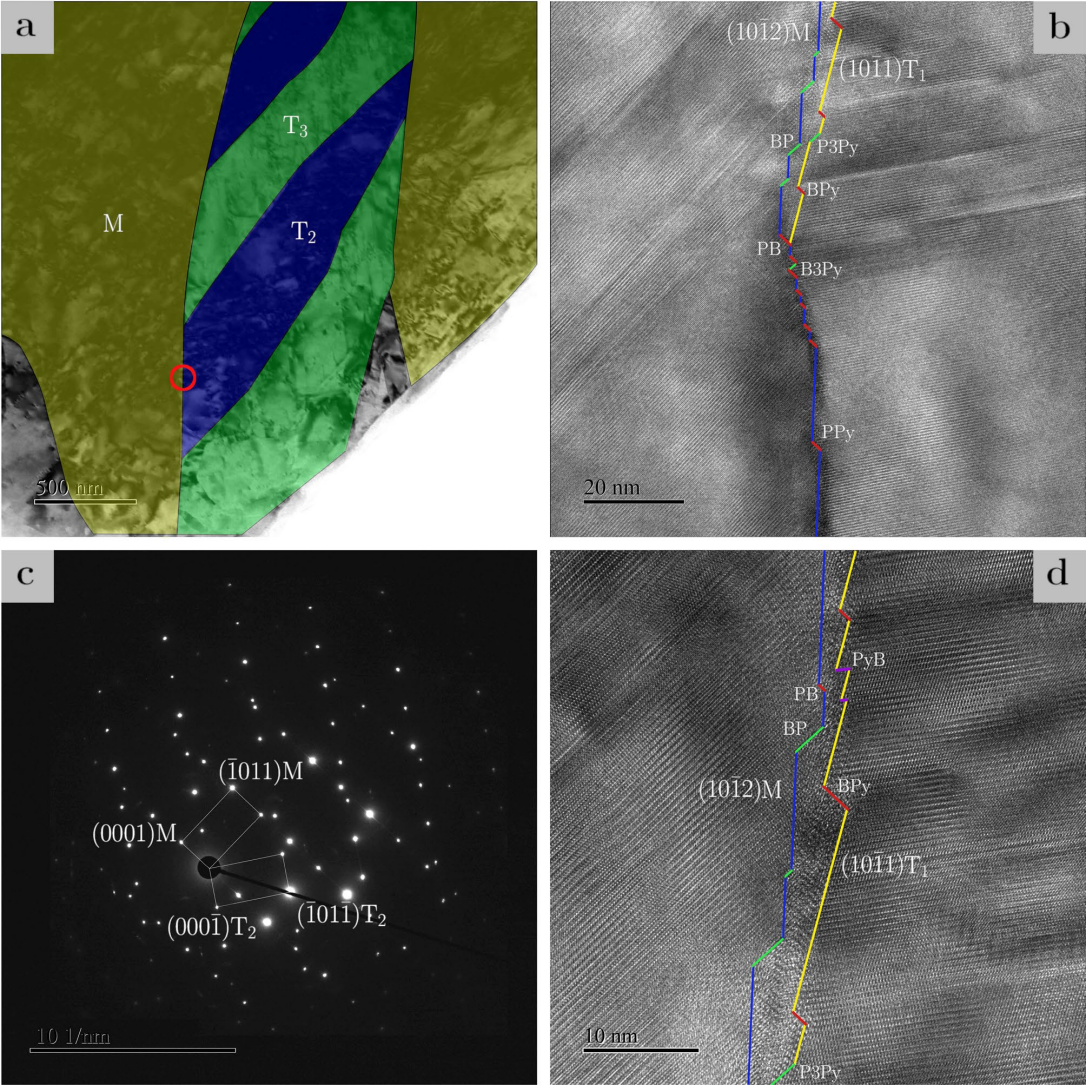
(**a**) Bright field image of the lower portion of the first area of interest, at low magnification; the matrix (M) is depicted in yellow, the secondary twin (${\textrm{T}}_2$) in blue, the tertiary twin (${\textrm{T}}_3$) in green. (**b**) HRTEM image of the region denoted by the red circle in (**a**); boundaries have been drawn using the following colour scheme. Blue: $(10\bar{1}2)$ plane of the matrix and primary twin; yellow: $(10\bar{1}1)$ plane of the primary and secondary twins; red: PB facets of the primary twin, BPy facets of the secondary twin and PPy facets of the double twin; green: BP facets of the primary twin, P3Py facets of the secondary twin and B3Py facets of the double twin; purple: PyB facets of the secondary twin. (**c**) Indexed SAD pattern of the region denoted by the red circle in (**a**). (**d**) HRTEM image of the upper region of (**b**), higher magnification, colour coding as in (**b**).

**Figure 9:**
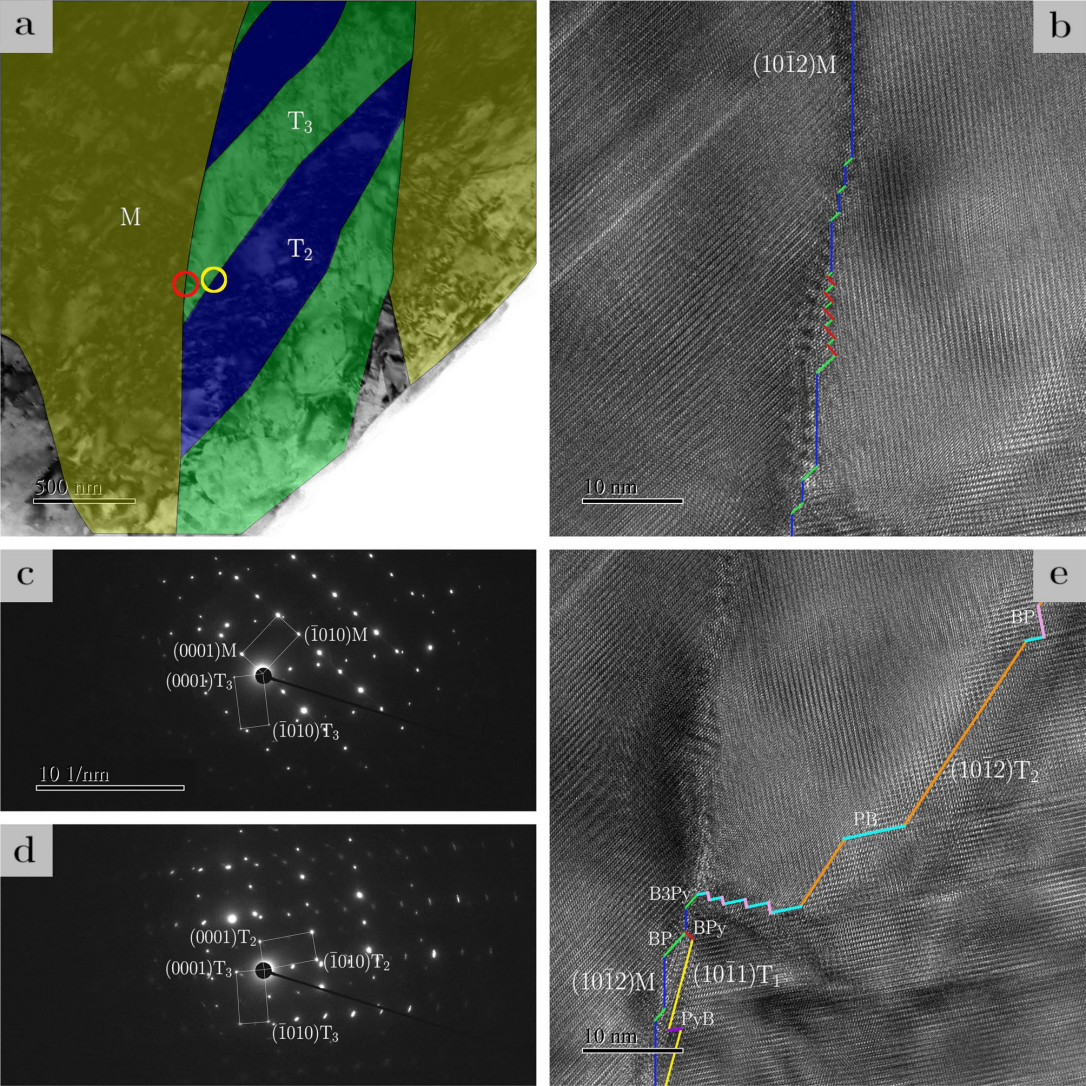
(**a**) Bright field image of the lower portion of the first area of interest, at low magnification; the matrix (M) is depicted in yellow, the secondary twin (${\textrm{T}}_2$) in blue, the tertiary twin (${\textrm{T}}_3$) in green. (**b**) HRTEM image of the region denoted by the red circle in (**a**), periodic contrast in the centre we interpret as moiré patterning; boundaries have been drawn using the following colour scheme. Blue: $(10\bar{1}2)$ plane of the matrix and primary twin; green: B plane of the matrix; red: P plane of the matrix. It should be noted that facets in the triple twin boundary are incommensurate, such that B and P planes of the matrix are not coincident with any rational planes of the triple twins. (**c**) Indexed SAD pattern of the region denoted by the red circle in (**a**). (**d**) Indexed SAD pattern of the region denoted by the yellow circle in (**a**). (**e**) HRTEM image of the junction between matrix, triple twin and double twin, directly below (**b**). Colour scheme is as follows. Blue: $(10\bar{1}2)$ plane of the matrix and primary twin; yellow: $(10\bar{1}1)$ plane of the primary and secondary twins; red: BPy facets of the secondary twin; green: BP facets of the primary twin and B3Py facets of the double twin; purple: PyB facets of the secondary twin; orange: $(10\bar{1}2)$ plane of the secondary and tertiary twin; aquamarine: PB facets of the tertiary twin; pink: BP facets of the tertiary twin.

TEM observations of the samples revealed the presence of twin-like objects in most grains. These appeared both as single twins as well as clusters of twins in several occasions. Two areas of interest were selected for in-depth investigation, Fig. 7. The first area, Fig. 7a, contains a long narrow twin, traversed by further internal twin boundaries. The SAD patterns taken at the boundary between the different regions of the twin and the matrix show misorientations of $38^{\circ }$ and $50^{\circ }$ about the $[1\bar{2}10]$ zone axis (Figs. 8c and 9c); the internal boundary separates two regions with misorientation $86^{\circ }$ $[1\bar{2}10]$ (Fig. 9d). The $38^{\circ }$ misorientation is consistent with either a C–T or T–C type 1 double twin, and is labelled ${\textrm{T}}_2$; however, as the internal boundary is identified as a $(10\bar{1}2)$ tension twin boundary and the $50^{\circ }$ misorientation area can be traced back to a T-C-T type 1a triple twin ($48^{\circ }$ misorientation, Table 1), labelled ${\textrm{T}}_3$, one can surmise that the ${\textrm{T}}_2$ region is a T–C double twin. This is confirmed upon investigation of HRTEM images: part of the primary tension twin is revealed to be left in the double twin boundary, Fig. 8b–d, with the primary ${\textrm{K}}_1$ plane being $(10\bar{1}2)$ and the secondary twinning occurring on $(10\bar{1}1)$ of the primary twin; the double twin finally retwins on its $(10\bar{1}2)$ plane, producing a T-C-T type 1a triple twin. These misorientation relations were displayed in SAD patterns of interfaces taken along the length of the twin, with alternating regions of double and triple twin; these have been coloured accordingly in the bright field images 8a and 9a, where yellow has been used to indicate the matrix, blue the double twin and green the triple twin. No sizeable portions of the primary tension twin (${\textrm{T}}_1$) appeared to be left, other than the slivers visible in the high-resolution images.

HRTEM images also reveal the presence of facets in all the boundaries analysed in the first area of interest. Figures 8b-d show how the BP and PB facets of the primary tension twin combine respectively with the P3Py and BPy facets of the internal secondary twin boundary to produce B3Py and PPy facets in the double twin boundary, as predicted in the theory section of this paper, while segments of the original $(10\bar{1}2)$ primary twin boundary remain largely unchanged (the reader is reminded that in this section the order in which the planes are listed in the facet names is that used in the HRTEM images). Conversely, no commensurate facets are present in the triple twin boundary, as shown in HRTEM image 9b, where the facets are along the B (green) and P (red) planes of the matrix but are not parallel to any rational plane of the triple twin. Hence, the triple twin boundary is fully incommensurate. Figure 9e depicts the junction between the triple twin and the double twin; BP and PB facets are observed in the internal $(10\bar{1}2)$ tertiary twin boundary, and a concentration of steps can be seen at the junction with the double twin and the matrix, indicating a high density of tertiary twinning disconnections.

**Figure 10:**
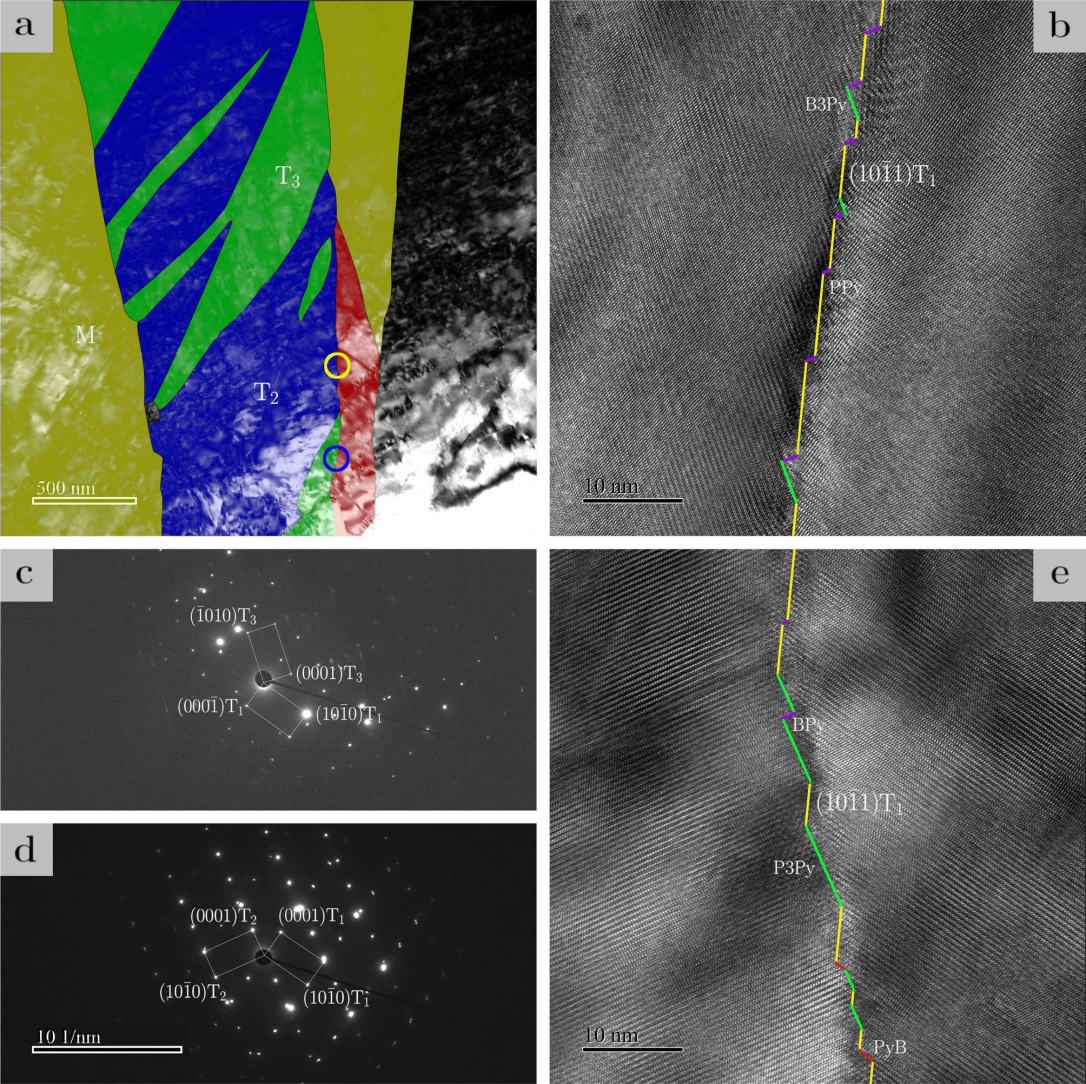
(**a**) Bright field image of the second area of interest, at low magnification; the matrix (M) is depicted in yellow, the primary twin (${\textrm{T}}_1$) in red, the secondary twin (${\textrm{T}}_2$) in blue, the tertiary twin (${\textrm{T}}_3$) in green. (**b**) HRTEM image of the region denoted by the blue circle in (**a**); boundaries have been drawn using the following colour scheme. Yellow: $(10\bar{1}1)$ plane of the matrix and primary twin; green: B3Py facets of the double twin; purple: PPy facets of the double twin. (**c**) Indexed SAD pattern of the region denoted by the blue circle in (**a**). (**d**) Indexed SAD pattern of the region denoted by the yellow circle in (**a**). (**e**) HRTEM image of the region denoted by the yellow circle in (**a**); boundaries have been drawn using the following colour scheme. Yellow: $(10\bar{1}1)$ plane of the matrix and primary twin; red: PyB facets of the primary twin; green: P3Py facets of the primary twin; purple: BPy facets of the primary twin; red: PyB facets of the primary twin.

The second area of interest is a cluster of twins, figure 7b. Upon investigation of the SAD patterns taken along interfaces, it was concluded that the cluster is made of bands containing all three twinning stages in the T-C-T type 1a sequence, interspersed with sections of matrix, i.e. material with similar orientation to that on either end of the cluster. A colour coding scheme similar to that of the previous area of interest has been used to aid the reader in interpreting the bright field image 10a: yellow: matrix; red: primary tension twin; blue: double T–C type 1 twin; green: triple T-C-T type 1a twin. One can note that many of the boundaries separating different regions of this object are not straight, and appear to be curved; this is once again due to faceting. In order to illustrate this point, HRTEM images were taken around the lower left of the cluster, where proximity to the electropolishing hole ensures optimal thickness of the sample to obtain better resolution.

One of these regions, Fig. 10a, is useful to shed some light on facets in a C–T double twin boundary. In this area, all twinning stages are found: primary tension twinning, secondary compression twinning and tertiary tension twinning. We focus on two boundaries: that between the primary and secondary twin, i.e. a compression twin boundary along $(10\bar{1}1)$ of the primary twin, and that between the primary and tertiary twin, i.e. a double C–T type 1 twin boundary, as the tertiary twinning occurs on $(10\bar{1}2)$ of the secondary twin. This allows us to examine the interface of a C–T double twin, as opposed to the T–C double twin boundary analysed in the first twin-like object.

The SAD pattern of the interface between the primary and secondary twin (Fig. 10d) displays a $58^{\circ }$ misorientation between the two sides of the crystal, thus identifying the secondary twin as a compression twin. A HRTEM image of the boundary confirms this, and shows that much of the boundary is made up of facets, specifically BPy, PyB and P3Py facets. In particular, the length of the P3Py facets is comparable to that of the segments of $(10\bar{1}1)$ boundary, such that the overall orientation of the boundary deviates significantly from $(10\bar{1}1)$. Shorter facets are observed in the double twin boundary. The SAD pattern of the interface between primary and tertiary twin displays a misorientation of $35^{\circ }$, which is close to the $38^{\circ }$ misorientation of C–T type 1 double twins. Upon observation of the HRTEM image, Fig. 10e, segments of the secondary $(10\bar{1}1)$ twin boundary are discovered, interspersed with B3Py and PPy facets, as anticipated in the theory section of this paper (there referred to as 3PyB and PyP, obeying the convention of listing the plane of the matrix first and that of the double twin second). Once again, the boundary macroscopically deviates from $(10\bar{1}1)$. Moreover, although the boundaries in Fig. 10b–e have considerably different orientations, the boundary between primary and secondary twin and that between primary and tertiary twin in the bright field image appear to have similar orientations, suggesting that the large deviation from $(10\bar{1}1)$ observed in Fig. 10e is a local phenomenon attributable to longer facets.

## Discussion

While double twins, mostly with C–T twinning sequence, are often reported in the literature, this is predominantly in the context of electron back-scatter diffraction (EBSD) experiments, which allow only to determine the relative orientation of neighbouring grains, i.e. the misorientation relations. Unless a twin has only partially retwinned, it is not possible to say with absolute certainty whether a double twin with misorientation $38^{\circ } \langle 1\bar{2}10\rangle$ is a tension-compression or compression-tension double twin; in addition, this misorientation can also be attributed to an interface between a tension and a compression twin, such that the shape of the twin-like objects and their position has to be taken into account to identify them properly.

These limitations are eliminated in TEM experiments, where analysis of SAD patterns enables a thorough investigation of the ${\textrm{K}}_1$ planes and therefore of the sequence of twinning events. The quality of the observations comes at the expense of quantity: TEM samples are necessarily small and have to be thinned and carefully prepared; imaged areas can span only a few hundred microns. Hence, not many TEM studies of double twin boundaries in magnesium and its alloys have been published; the most extensive to date was conducted by Cizek and Barnett^7^, who analysed 58 twin-like objects in a commercial Mg alloy, identifying a high proportion of these as C–T double twins and measuring their matrix to double twin interface planes. This study could not, however, identify the cause of the deviation of double twin boundaries from the primary ${\textrm{K}}_1$ planes. Conversely, in the present work, we were able to obtain high-resolution TEM images, which allow us to identify facets in the single and double twin boundaries with C–T and T–C twinning sequences, thus characterising them fully.

A C–T double twin boundary was imaged using HRTEM in a study by Lentz et al.^44^, although a small residue of the primary twin was left between the secondary twin and the matrix, leading the authors to state that double twin boundaries always contain such a residue and that direct interfaces between the double twin and the matrix are not observed. The present study contradicts their argument: Fig. 8b shows clearly that while in the upper half of the interface there is a fraction of primary tension twin left between the secondary twin and the matrix, none of the primary twin is found in the lower half, where the interface is directly between the double twin and the matrix. This is evidenced by the commensurate facets formed in the double twin boundary, consistent with the combination of primary and secondary twinning disconnections envisaged in the theoretical analysis given above. Hence, high-resolution images allow us for the first time to shed a light on the morphology of double twin boundaries.

In particular, we are able to conclude that the orientation of the double twin interface is largely predetermined by that of the primary twin, i. e. $(10\bar{1}2)$ for T–C double twins and $(10\bar{1}1)$ for C–T double twins. The primary twin to matrix boundary itself however contains facets, such that it is likely, on average, to deviate from the primary ${\textrm{K}}_1$ plane; these facets are retained in the double twin boundary and remain commensurate. In light of these observations, it is not surprising that when measuring the orientation of single and double twin boundaries, the results are spread around the pole corresponding to the ${\textrm{K}}_1$ plane of the primary twin. Starting with T–C double twins, in the first area of interest, Fig. 7a, the boundary between the double twin and the matrix, though curved, is approximately aligned with $(20\bar{2}5)$; this is $\sim 6^{\circ }$ from the primary ${\textrm{K}}_1$ plane, $(10\bar{1}2)$. In the second area of interest, Fig. 7b, the orientation of four T–C double twin boundaries in the cluster is measured; it is evident that these interfaces are considerably curved, but the average orientations are approximately along $(20\bar{2}5)$ , $(10\bar{1}4)$ ($\sim 18^{\circ }$ from $(10\bar{1}2)$) and $(10\bar{1}3)$ ($\sim 11^{\circ }$ from $(10\bar{1}2)$). Due to the presence of portions of the primary twin in parts of the cluster, e.g. the region considered in Fig. 10a, it is also possible to measure the orientation of a C–T double twin boundary as approximately $(40\bar{4}5)$; this is $\sim 6^{\circ }$ from $(10\bar{1}1)$. However, it is observed that the compression twin boundary itself deviates from $(10\bar{1}1)$, and is more closely aligned to $(20\bar{2}3)$; indeed, long facets could be seen in a HRTEM image of this boundary, Fig. 10e. This is worthy of note because Cizek and Barnett^7^ reported that a fraction of the double twin boundaries they analysed were aligned with the $(20\bar{2}3)$ plane: it is possible that this was already the macroscopic orientation of the primary twin interface.

The present study includes the first high-resolution observation of T–C double twin boundaries in a magnesium alloy. This double twinning mode is only reported in Mg in the context of hierarchical twinning, where thin secondary compression twins are formed inside large primary tension twins and are not observed to reach the primary twin boundaries. The only observation of T–C double twins surrounded by untwinned material in a hcp metal was reported in a cold rolled Zr alloy by An et al.^12^. Their HRTEM investigation reveals the presence of faceted primary tension twin boundaries, as well as faceted double twin boundaries; the kinds of facets are the same as those identified in the current work.

Rarer than double twins are triple twins, which are scarcely reported. In particular, a review of the available literature shows that T-C-T triple twins are referred to in which the primary tension twin has grown so large as to consume a sizeable portion of the grain, when not its entirety. Secondary compression twins are then found to nucleate within the primary twins, albeit remaining of limited size and shaped into thin lamellae, as tertiary tension twinning is activated inside them effectively stifling further growth. Therefore, in these cases, the tertiary twin is wholly contained within the primary twin, and only interfaces between the primary and tertiary twin are observed, indistinguishable from boundaries of C–T double twins with the matrix.

The triple twins captured in the work here presented differ from those in the literature in that they interface directly to the matrix. In all analysed areas of our sample, the primary tension twins were never found to have grown beyond a few microns before retwinning twice, leading to the $\sim 48^{\circ }$ misorientation with the matrix characterising triple twins. Indeed, barely any thin residues of primary twin are left in the twin investigated in Fig. 8, and only narrow portions are found in the cluster in Fig. 7b. The secondary compression twin consumes the tension twin in most places; finally, the tertiary tension twin grows within the secondary twin, forming several bands along the length of the twin but not fully retwinning it. Nor does the tertiary twinning event appear to change the orientation of the macroscopic double twin boundary significantly; this is in agreement with the proposed theory, as no new kinds of facets can be created in the triple twin boundary that did not already exist in the double twin.

Several reasons can be proposed for the fundamental difference in the morphology of the triple twins observed here in contrast with those reported in the literature. The first concerns the alloy composition. In the published literature, non-rare-earth-containing alloys were used: Mg–3.3Al–1Zn^41,43,46^, Mg–4Li^44^, pure Mg^42^, Mg–2.6Al–0.9Zn–0.37Mn^45^. The alloy used in this study contains Nd, with a large atomic radius compared to Mg, and which has been reported to segregate to twin boundaries^47^ possibly inhibiting twin growth and favouring facet formation. Moreover, in the present experiment the samples were hot rolled, as opposed to warm rolling^41,43^ and room temperature tensile testing^42,44–46^. STEM-EDS mapping of the region containing the cluster of twins in the present study indicates no segregation of Al or Nd to twin boundaries, and in particular Nd was found to be below the detection threshold. A high density of Nd–Al precipitates was observed in SEM investigations of the samples, and precipitates have been found to inhibit tension twin growth in Mg–Al–Zn and Mg–Zn alloys^48^, opposing the motion of twinning disconnections and discouraging further twin growth even after they have been engulfed by the twin^49^. The density of precipitates in the alloy in consideration in this study is not such that it could explain the limited growth of all the twins in the sample, but precipitates are likely to be a contributing factor, while solute segregation at twin boundaries is not expected to have played a role in the microstructures studied here.

## Conclusion

In this article a disconnection-based model for facet formation in single and double twin boundaries in hcp crystals was presented. Commensurate facets formed in single twin boundaries by primary twinning disconnections were shown to be transformed into commensurate facets of the double twin by the action of secondary twinning disconnections; the model was applied to both tension-compression and compression-tension double twins, i.e. $\{10\bar{1}2\}-\{10\bar{1}1\}$ and $\{10\bar{1}1\}-\{10\bar{1}2\}$ double twins respectively. The potential for commensurate facets in triple twin boundaries with tension-compression-tension twinning sequence was also considered, and it was found that tertiary twinning disconnections cannot in this case transform facets of the double twin into commensurate facets of the triple twin. Theoretical predictions were then tested through transmission electron microscopy. Twin-like objects found in a hot rolled Mg-1.18wt%Al-1.77wt%Nd alloy were investigated, and SAD patterns of the interfaces revealed the presence of tension twins, double tension-compression twins and triple tension-compression-tension twins. High-resolution TEM was used to capture images of the single, double and triple twin interfaces, evidencing the presence of commensurate facets in the former two and incommensurate facets in the latter, as expected from theoretical considerations. In single twins, terraces of commensurate twin boundary were found to be separated by facets; in double twins, terraces of the original primary twin boundary, now incommensurate, were interspersed with commensurate facets; in triple twins, the interface was made up of incommensurate terraces and facets. It was concluded that deviations of the interface from the primary ${\textrm{K}}_1$ plane observed in single, double and triple twins can be attributed to faceting.

Therefore, the main findings of this study are the following.

- Faceted single twin boundaries in magnesium are fully commensurate. Facets are formed as a consequence of twinning disconnection pileup in both tension and compression twins. Existing facets in single twin boundaries combine with facets of the secondary twin, thus producing commensurate facets in the matrix/double twin interface, whilst leftover segments of boundary parallel to the primary ${\textrm{K}}_1$ plane are made incommensurate. T-C-T triple twin to matrix interfaces are fully incommensurate, as no facets of the tertiary twin combine with facets of the double twin.
- Single, double and triple twin boundaries are observed via HRTEM. Facets contained in the interfaces conform to theoretical predictions, and are found to be the cause of the deviation of the interfaces from the primary ${\textrm{K}}_1$ planes. Notably, interfaces between T–C double twins and the matrix, as well as between T-C-T triple twins and the matrix, are observed for the first time in a magnesium alloy.

## Data Availability

All data generated or analysed during this study are included in this published article.
